# Deciphering the ATP-binding mechanism(s) in NLRP-NACHT 3D models using structural bioinformatics approaches

**DOI:** 10.1371/journal.pone.0209420

**Published:** 2018-12-20

**Authors:** Jitendra Maharana, Debashis Panda, Sachinandan De

**Affiliations:** 1 Department of Bioinformatics, Orissa University of Agriculture and Technology, Bhubaneswar, Odisha, India; 2 Distributed Information Centre (DIC), Department of Agricultural Biotechnology, Assam Agricultural University, Jorhat, Assam, India; 3 Animal Genomics Lab., Animal Biotechnology Centre, ICAR-National Dairy Research Institute, Karnal, Haryana, India; Universitat Hohenheim, GERMANY

## Abstract

Nucleotide-binding and oligomerization domain (NOD)-like receptors (NLRs), the first line of defense, are the cytosolic pattern recognition receptors (PRRs) that regulate the inflammatory activity in response to invading pathogens. NLRs are the members of AAA+ ATPase superfamily that comprises of N-terminal EBD(s), a centrally positioned NOD/NACHT and varying range of LRRs towards the C-terminal end. Due to the lack of structural data, the functional aspects of NLRP-signaling mechanism, which includes pathogen recognition, nucleotide-binding, and sensor-adaptor-effector interactions, are not fully understood. In this study, we implemented structural bioinformatics approaches including protein modeling, docking, and molecular dynamics simulations to explore the structural-dynamic features of ADP-/ATP-Mg^2+^ binding in NLRP^NACHT^ models. Our results indicate a similar mode of ATP-Mg^2+^ binding in all NLRP^NACHT^ models and the interacting residues are found consistent with reported mutagenesis data. Accompanied by the key amino acids (proposed to be crucial for ATP-Mg^2+^ coordination), we further have noticed that some additional conserved residues (including ‘Trp’ of the PhhCW motif, and ‘Phe’ and ‘Tyr’ of the GFxxxxRxxYF motif) are potentially interacting with ATP during dynamics; which require further experimentation for legitimacy. Overall, this study will help in understanding the ADP-/ATP-Mg^2+^ binding mechanisms in NLRPs in a broader perspective and the proposed ATP-binding pocket will aid in designing novel inhibitors for the regulation of inflammasome activity.

## Introduction

Innate immunity constitutes the first line of defense against infectious pathogens. It is governed by several sets of germ-line encoded receptors, known as pattern recognition receptors (PRRs). These receptors mediate the host-defense mechanism against typical patterns, namely pathogen-associated molecular patterns (PAMPs) or host-derived danger-associated molecular patterns (DAMPs) [1]. The PRRs encompass five major families: Toll-like receptors (TLRs), nucleotide-binding and oligomerization domain (NOD)-like receptors (NLRs), retinoic acid-inducible gene (RIG)-I like receptors (RLRs), C-type lectin receptors (CLRs) and absent in melanoma (AIM)-2 like receptors (ALRs). Of these, NLRs, RLRs, and ALRs are restricted to the cytoplasm, while TLRs and CLRs are membrane-bound [2]. NLRs are evolutionarily conserved PRRs, play fundamental roles both in plant and animal host-defense mechanism and are classified under the STAND (signal transduction ATPase with numerous domains) sub-clade of AAA+ ATPase superfamily [3,4]. Structurally, NLRs show tripartite domain architecture; diverse N-terminal effector binding domain (EBD), centrally positioned NOD/NACHT and variable numbers of LRRs towards the C-terminal end. A total of 22 and 34 members of NLRs have been reported in human and mice, respectively. These receptors are classified into five major sub-groups according to their amino-terminal domain distribution; NLRA (CIITA), NLRB (NAIP), NLRC (NLRC1-5), NLRP (NLRP1-14) and NLRX (NLRX1) [5]. Apart from their role in the host-defense mechanism, most of the NLRs regulate gametogenesis [6–9].

The PYrin Domain (PYD) containing NLRs are evolutionarily highly conserved and show tripartite domain architecture (PYD-NACHT-LRRs). In this sub-family, NLRP1 and NLRP10 are two unique members display distinct type of domain architecture; the former one shows two additional domains (FIIND and CARD) at the C-terminal end, and the later one is devoid of LRRs. In cytosolic environment, the NLRPs are found in idle state before pathogenic invasion, and the recognition of PAMPs/DAMPs activates the receptors. This PAMP/DAMP-recognition, which is mostly driven by LRR domains, induces the conformational reorientation and is thought to facilitate the ATP-dependent self-oligomerization of the receptors [10,11]. These self-oligomerized active NLRPs in association with adaptor protein ASC (apoptosis associated speck-like protein containing CARD) and effector molecule procaspase-1, orchestrate the multi-protein caspase-1-activating complexes called Inflammasome. This assembled multi-protein signaling complex leads to the maturation and release of pro-inflammatory cytokines (IL-1β and IL-18), and often induction of cell death (apoptosis and pyroptosis). In 2002, Martinon et al. reported NLRP1 as the first receptor to assemble inflammasome in association with ASC and procaspase-1 [12]. Later, several NLRs (NLRP3, NLRP7, NLRP12 and CARD-containing NLRC4) along with two non-NLR proteins (AIM2 and IFI16) were reported to assemble inflammasome [12–14].

Dysregulation in NLRP-gene function due to polymorphisms and mutations has been observed to be associated with several auto-inflammatory, autoimmune as well as metabolic diseases. For instance, the polymorphisms in NLRP1 are linked to a variety of autoimmune diseases viz., Addison’s diseases, autoimmune thyroid diseases, giant cell arteritis, Alzheimer’s diseases [15–18], etc.; NLRP2 is associated with arsenic-induced skin lesions and familial imprinting disorder [19,20]. SNPs in NLRP3 are linked to Type-I diabetes, celiac disease, and psoriasis [21,22]. Apart from this, several mutations in NLRP3 are also linked to familial cold autoinflammatory syndrome 1 (FCAS1), cryopyrin-associated periodic fever syndrome (CAPS), type-II diabetes and atherosclerosis [23–25]. It has been observed that NLRP5 is associated with reproductive and multilocus imprinting disorders [7], while dysregulation in NLRP6 is linked to colitis-induced tumorigenesis [26]. Additionally, the mutations in NLRP12 are associated with hereditary periodic syndrome [27]. Moreover, several reports on NLRP2 and NLRP7 notified their association with recurrent miscarriage (abnormal human pregnancies and embryonic development) [6, 9, 28]. And, the reports on NLRP14 gene function indicated its regulatory role in spermatogenesis [8,9].

Despite emerging shreds of evidence, various aspects of NLR-signaling mechanisms such as pathogen-recognition, nucleotide binding, and protein-protein interactions, etc. are yet to be deciphered. In NLR family, NLRP is the largest subfamily, plays essential roles both in mammalian innate immune and reproductive systems. Due to the lack of structural data, it remains a challenge to understand the molecular basis of NLRP signaling mechanism. Henceforth, to elucidate the mode and nature of ADP/ATP binding, and their role in switching mechanism of NLRPs, we adopted the structural bioinformatics approaches. The results from this computational investigation revealed similar mode of ADP/ATP-binding in all NLRP^NACHT^ models. But, no switching mechanism of NACHT domains was observed during the course of simulation time in response to ADP-/ATP-Mg^2+^. In all, our study would be helpful in understanding the mode and mechanisms of ADP-/ATP-Mg^2+^ binding in NLRP^NACHT^ domains and will show future directions to develop new therapeutics for the patients suffering from acute and chronic diseases.

## Computational methods

### Identification of functional domains

The primary protein sequences of human NLRPs (NLRP1-14) were retrieved from NCBI protein database (S1 Table). The individual domains, sub-domains and motif regions were acquired from UniProt (www.uniprot.org) and literature studies [10,29]. In addition, the multiple sequence alignment of NLRP proteins was carried out using MAAFT [30] to probe the functional domain boundaries and nucleotide-binding motifs.

### Molecular modeling

After probing the functional domains and nucleotide-binding motifs, we considered the NACHT domains of NLRP1-14 for the 3D model building to infer the mode and nature of ADP/ATP-binding mechanism. First, the secondary structure prediction of NLRP^NACHT^ domains was carried out using PSIPRED [31]. Thereafter, suitable template(s) for individual NACHT domains was searched using DELTA-BLAST tool [32]. Due to low sequence identity with available templates, the 3D model building was performed using I-TASSER server [33]. Out of the generated models, the best model for each NLRP^NACHT^ domain was chosen based on the lowest CS-score, and refined using GalaxyRefine server [34]. Then, the refined models were validated using PROCHECK [35], ProSA [36], and Verify3D [37] web servers.

### Molecular docking

ATP-binding sites of the built NLRP1-14^NACHT^ models were anticipated from available literature [10,29] and sequence-structure analysis. To understand the mode and nature of ADP and ATP-binding, molecular docking simulations were performed in two different approaches. In the first approach, the docking set up was prepared using AutoDock tools [38], and the docking simulations were carried out in AutoDock Vina [39]. Docking parameters were acquired from the previous study [40]. In the second approach; the ADP, ATP and Mg^2+^ were placed in the active site pocket of all NLRP^NACHT^ models based on *Oc*NOD2 (5IRL [41]) and APAF1 (3JBT [42]) crystal structures. NLRP1-14^NACHT^ models were first superimposed with *Oc*NOD2 and APAF1 structures and later, the ADP-/ATP-Mg^2+^ coordinates were merged using PyMOL. After placing the ADP/ATP and Mg^2+^ in the nucleotide-binding site of all NLRP^NACHT^ models, the unrealistic close contacts (intermolecular bumps/steric clashes) between ADP/ATP and surrounding residues were carefully cleared using DSV 4.2 (BIOVIA, 2017). Thereupon, to infer the dynamic characteristics of NACHT domains in ADP-/ATP-Mg^2+^-bound state, all the complexes generated via AutoDock Vina and manual docking (along with Nlrc4^NACHT^-ADP-/ATP-Mg^2+^) were simulated for 50 ns timescale.

### MD simulation

MD simulations of apo and holo (ADP-/ATP-Mg^2+^-bound) systems were performed in GROMACS 5.1 simulation suite [43] using Amber ff99SB-ILDN force field [44] in the periodic boundary condition. The topology files for deprotonated ADP (-3e) and ATP (-4e) were generated using Antechamber tool v 1.11 (via CCPN web server) [45]. The simulation systems were solvated in TIP3P water model and neutralized by adding a physiological ionic strength (0.15 M) of NaCl. The energy minimization was performed using steepest descent algorithm until a tolerance of 1000 kJ mol^-1^ in order to avoid steric conflicts and high energy interactions between the atoms. The simulation systems were equilibrated for 0.1 and 1 ns under NVT (constant volume) and NPT (constant pressure) conditions, respectively. PME (particle mesh Ewald) summation method was used for the computation of long-range electrostatic interactions and temperature coupling was set to 300 K using modified Berendsen thermostat algorithm. After equilibrium, the final production simulations were run for 50 ns timescale (S2 Table).

### Analysis of MD trajectories

After MD simulation, the trajectory analysis was carried out using integrated modules of GROMACS and VMD [46]. Backbone root mean square deviation (RMSD) and total numbers of H-bonds (as a function of simulation time) were calculated using *gmx rms* and *gmx hbond* tools, respectively. To get the representative ADP-/ATP-Mg^2+^ bound NLRP^NACHT^ structures (for interaction study), we performed the cluster analysis of holo trajectories using *gmx cluster* tool. The interaction analysis of ADP-/ATP-Mg^2+^ bound representative structures was performed using PISA [47]. The structural and molecular illustrations were generated in PyMOL (academic license). The inter-residual distance(s) between ATP-Mg^2+^ and interacting residues were calculated using the *gmx mindist* program. Principal component analysis (PCA) was performed using *gmx covar* and *gmx anaeig* tools to understand the global motion of the proteins by calculating the eigenvalues and principal components (PC1 and PC2) of main-chain atoms. All the 2D graphs generated from trajectory analysis were prepared using Grace 5.1.23 (http://plasma-gate.weizmann.ac.il/Grace/).

## Results and discussion

### Functional domains, ADP/ATP-binding motifs and 3D models

**Fig 1:**
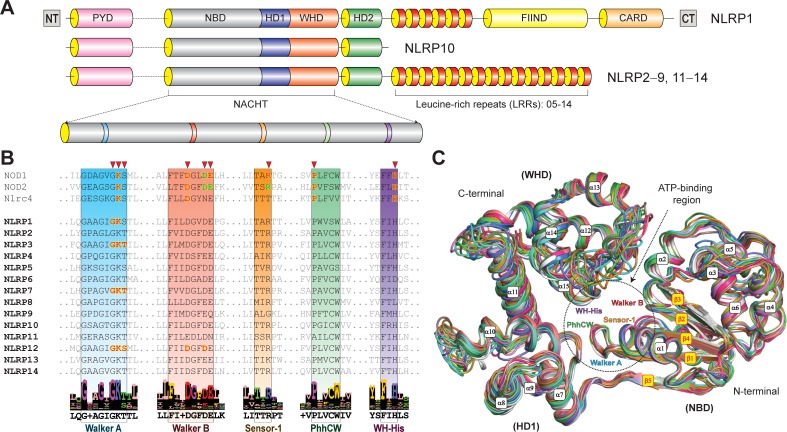
Domain organization, ATP-binding motifs and structural overview of NLRP^NACHT^ 3D models. **(A)** NLRPs show tripartite domain architecture (PYD-NACHT-LRR) except NLRP1 (which possesses two additional C-terminal domains: FIIND and CARD) and NLRP10 (that lacks the LRRs). Magnifying view of NACHT domain illustrates key ATP-binding motifs. **(B)** The key ATP-binding motifs (from multiple sequence alignment) are boxed in different colors (Walker A: blue; Walker B: salmon; Sensor- 1: orange; PhhCW motif: green; WH-His: purple). The experimentally validated amino acids involved in ATP-binding are shown in red fonts and those not interacting are displayed in green font. **(C)** Structural overview of NLRP^NACHT^ models based on *Oc*NOD2 crystal structure (5IRL [41]) (illustrated in different colored cartoons: NLRP1, white; NLRP2, deep salmon; NLRP3, marine; NLRP4, green; NLRP5, violet; NLRP6, orange; NLRP7, hot pink; NLRP8, slate; NLRP9, sand; NLRP10, cyan; NLRP11, dirty violet; NLRP12, forest; NLRP13, green-cyan; and NLRP14, olive) indicate the secondary structural elements (α-helices and β-sheets, which are labeled in white and red fonts within red and yellow boxes, respectively). The ATP/ADP-binding region is displayed in black dotted circle along with the NTP-binding motifs.

Domain analysis of NLRPs (1–14) revealed that most of the receptors are comprised of three functional domains; N-terminal PYD, central NACHT and variable numbers of LRRs at the C-terminal end. NLRP1 has two additional domains, FIIND and CARD towards the C-terminal region, whereas NLRP10 lacks the LRR (Fig 1A; S1 Table). Consistent with recently resolved Nlrc4 (4KXF) and *Oc*NOD2 (5IRL) structures [41,48] and sequence analysis results, we outlined three functional sub-domains of NACHT/NOD modules; (a) the nucleotide-binding domain (NDB) positioned after PYD, (b) the helical domain 1 (HD1) and (c) the winged helix domain (WHD) towards C-terminal end. Like Nlrc4/*Oc*NOD2, we also found the second helical domain (HD2) in all NLRP proteins residing between WHD and LRR (Fig 1A).

The sequence analysis of NLRP1-14^NACHT^ domains revealed five conserved nucleotide-binding motifs [Walker A (GxxxxGK[S/T]; where ‘x’ is any amino acid), Walker B [hLhhh(D/E); ‘h’ denotes any hydrophobic residue], Sensor 1 (LLhTxR), PhhCW motif (also termed as ‘GxP’ in other NTPase family members) and WH-His (FxH)], which are responsible for ATP-binding and coordination of Mg^2+^ ion (Fig 1B) [10]. To investigate the ADP-/ATP-Mg^2+^-binding mechanisms, we constructed 3D models of NLRP1-14^NACHT^ domains using I-TASSER server. The 3D structures with the lowest CS-score were refined and validated with their stereochemical parameters, and the model validation report (S3 Table) indicated their reliability. The structural analysis of NLRP^NACHT^ models revealed a similar spatial (3D) arrangement of structural subunits (NBD-HD1-WHD). The ‘NBD’ comprised of five parallel β-sheets (β1-β5) along with five α-helices (α1-α5), the HD1 exhibited four α-helices (α6-α9) and the C-terminal sub-domain, ‘WHD’ consists of four α-helices (α10-α13) and two β-sheets (β6-β7) (Fig 1C).

### Molecular docking of ATP

The molecular dockings were carried out (using AutoDock Vina) to identify a plausible mode of ADP/ATP-binding in NLRP1-14^NACHT^ models and the conformations having least binding energy (S4 Table) were taken for further studies. Till date, only two ADP-bound NLR structures have been resolved (Nlrc4 (4KXF) [48] and *Oc*NOD2 (5IRL) [41]) and both the structures share a common mode of nucleotide-binding. The docking analysis revealed a non-conserved pattern of ADP/ATP-binding in all the auto-docked systems and the binding conformations of the nucleotides were found different from those observed in Nlrc4 and *Oc*NOD2 structures (S1A Fig). Therefore, we adopted a manual approach of docking, where the γ-phosphate group of ATP was deeply buried inside the core cavity formed by NBD (Walker A, Walker B and Sensor 1 residues) and WHD, and adenine moiety was placed in between HD1 and NDB (see S1B Fig). Herein, to understand the dynamic stability of ADP/ATP in the binding cavity, MD simulations of all the ADP-/ATP-Mg^2+^-bound NLRP1-14^NACHT^ complexes (both auto-docked and manually-docked) were carried out for 50 ns timescale.

### Evaluation of ADP/ATP-NLRP^NACHT^ complex models

**Fig 2:**
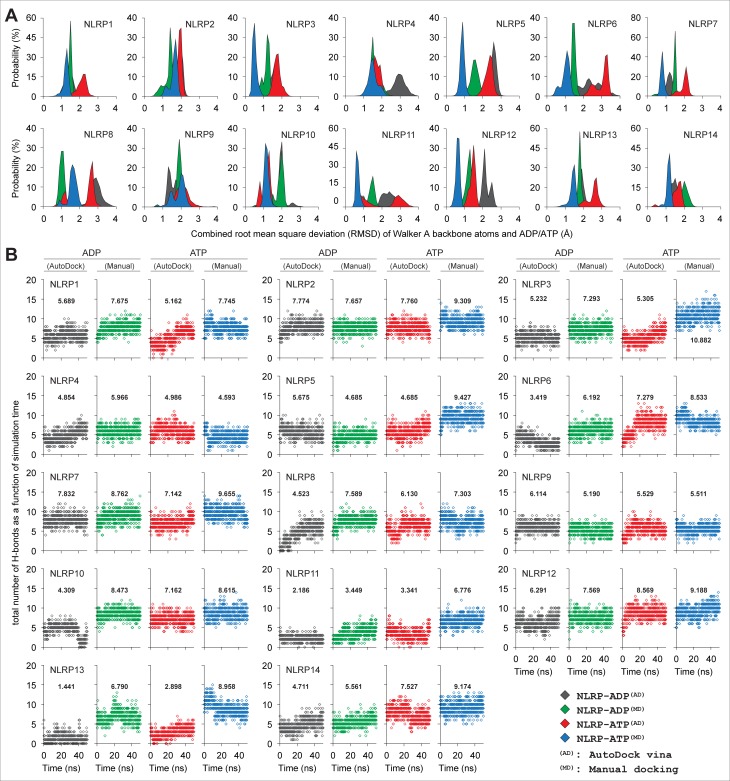
Analysis of NLRP^NACHT^-ATP/Mg^2+^ complex stability and intermolecular H-bonds formed. **(A)** Distribution of ADP/ATP-Walker A combined RMSD calculated from 50 ns trajectories (X-axis represents the RMSD values and Y-axis denotes the distribution probability). **(B)** Intermolecular H-bonds between ADP/ATP and NLRP^NACHT^ models of individual complexes, calculated from respective trajectories as a function of simulation time. The total numbers of H-bonds are depicted within the graphs.

The experimental evidence suggested that Walker A (GxxxxGK[S/T]) motif of AAA+ ATPase proteins is crucial for nucleotide binding and particularly, the conserved ‘Lys’ coordinates the β/γ-phosphate group of nucleotide [48–53]. Therefore, to assess the stability of the trajectories of ADP-/ATP-Mg^2+^-bound NLRP^NACHT^ complexes out of the auto-docked and manually-docked systems, we calculated the combined RMSD of Walker A backbone atoms and ADP/ATP. As displayed (in Fig 2A), we noticed a comparatively lower and stable array of RMSD in manually-docked systems; however, in auto-docked systems, the higher and unstable-deviations were observed. To understand the binding stability between ADP/ATP and NLRP^NACHT^ models, the total numbers of intermolecular H-bonds were analyzed as a function of simulation time. Herein, we observed comparatively higher numbers of H-bonds in the manually-docked systems with some exceptions, which include ATP-bound NLRP4, ADP-bound NLRP5, and ADP/ATP-bound NLRP9 (where we found comparatively lesser H-bonds in manually-docked complexes) (Fig 2B). Further, to evaluate the dynamic-stability of ADP/ATP’s orientation in binding cavity, we superimposed the pre- and post-MD snapshots. As displayed (see S2 Fig), stable orientations of ADP/ATP were noticed in most of the manually-docked complexes (except ATP-bound NLRP8); by contrast, drastic changes in ADP/ATP-binding orientations were observed in auto-docked complexes. Thus, based on the above observations, the manually-docked trajectories were chosen for interaction analysis.

### NLRP^NACHT^ models exhibited a similar mode of ATP-binding

**Fig 3:**
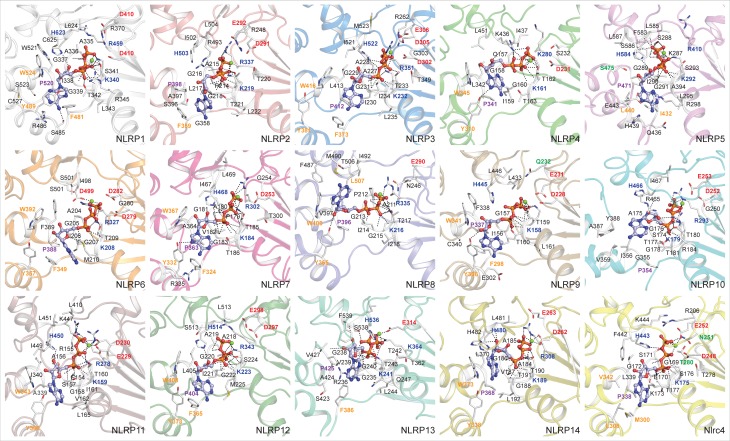
Overview of detailed intermolecular interactions governed by ATP and NLRP^NACHT^ models. The NLRP^NACHT^ models are displayed in cartoon; ATPs are visualized in light blue ball-sticks and the residues interacting within 5Å of ATP are visualized in white stick model. The black dotted lines represent the intermolecular polar contacts. The key interacting residues (of proposed ATP-binding motifs) are presented in bold font and colored based on their physicochemical parameters.

**Fig 4:**
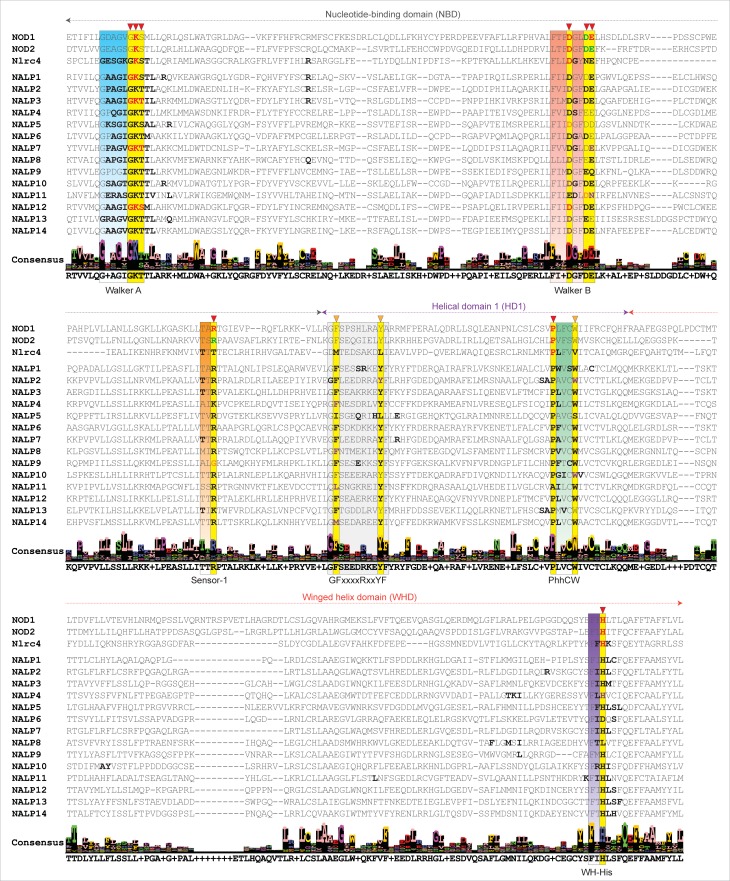
Multiple sequence alignment of NACHT domains of NLRPs, NOD1, NOD2 and Nlrc4; the residues interacting with ATP are displayed in bold fonts. The critical ATP-binding residues (proved experimentally), presented in red-bold font; the residues found within 5-6Å of ATP (adopted from atomic/residual distance calculation; Fig 4) are visualized in black fonts. The sequence motifs crucial for ATP binding are boxed in different colors (Walker A: blue; Walker B: salmon; Sensor 1: orange; GFxxxxRxxYF motif: light gray; PhhCW motif: green; WH-His: purple). The key ATP-coordinating residues are highlighted in yellow boxes and are marked in inverted triangle.

The NTP-binding modes in Walker A (P-loop) motif-containing proteins are similar [49]. Here, to determine the modes of ATP-binding in dynamically stable complexes, the average snapshots of NLRP1-14/Nlrc4^NACHT^-ATP-Mg^2+^ conformations (obtained from cluster analysis) were considered for interaction analysis. As visualized (in Fig 3), we observed a similar mode of ATP-binding in NLRP^NACHT^ models as that of ADP’s orientation in *Oc*NOD2/Nlrc4 solution structures [41,48]. It was proposed that the key residues, ‘Lys’ (Walker A), ‘Asp’ (first acidic residue of Walker B; which coordinates the Mg^2+^), ‘Arg’ (Sensor 1), ‘Pro’ (PhhCW motif) and ‘His’ (WH-His) were essential for nucleotide binding in NLRs [10,29]. Hence, to identify the conserved residues that are potentially contributing to ATP-binding, first, we carried out the interaction analysis using PISA program and visualized the interacting residues along with ATP and Mg^2+^ (Fig 3). To observe the involvement of key conserved residues in the said interaction, all the interacting residues were outlined in the alignment file (Fig 4). Herein, we noticed some of the conserved residues are not interacting with ATP in representative structures. To understand the involvement of the conserved residues distant by more than 5Å from the nucleotide, we computed their dynamic-distance (between distant-residues and ATP) as a function of simulation time and further confirmed their participation in ATP-binding.

The interaction analysis revealed five-seven polar contacts between the Walker A motif and γ-phosphate of ATP. Specifically, the conserved ‘Lys’ of the Walker A motif and ‘Arg’ of Sensor 1 (‘Lys’ in NLRP4 and NLRP13, and excluding ‘Gly’ of NLRP9) formed four-six numbers of H-bonds with the γ-phosphate group of ATP (Fig 4). The molecular interaction combined with distance calculation results (S3 Fig) revealed that the first acidic residue ‘Asp’ (‘Glu’ in NLRP11) of the Walker B motif was found within 5Å of ATP-Mg^2+^; however, the interaction (within 6Å of ATP) of second and third (‘Q’ in NLRP9 and ‘N’ in NLRP11) acidic residues (of Extended-Walker B) was found to be transient. The conserved ‘Pro’ of the PhhCW motif (along with ‘Trp’ (‘Ser’ in NLRP5)) was found to form hydrophobic contacts with ribose sugar of ATP (Fig 3). Furthermore, we observed strong hydrophobic contacts and/or electrostatic interactions between the adenine ring of ATP and conserved ‘Phe’ and ‘Tyr’ of GFxxxxRxxYF motif (identified in this study), suggesting their significant contribution towards ATP-binding (Figs 3, 4). Moreover, the conserved ‘His’ of WHD (WH-His), which was proposed to be crucial for the auto-inhibition mechanism in Nlrc4 [48], was found to form polar/electrostatic contacts with γ-phosphate in all the NLRP1-14^NACHT^-ATP-Mg^2+^ complexes (Fig 4). Apart from these, we also noticed some deformities in interaction pattern in NLRP4 (no interaction of second-third residues of Extended-Walker B motif), NLRP5 (no interaction with Walker B residues), NLRP10 (no participation of ‘Phe’ and ‘Tyr’ of GFxxxxRxxYF motif) and NLRP13 (no participation of conserved ‘Tyr’ of GFxxxxRxxYF motif in the interaction), which might be due to large structural changes during dynamics. In addition to ATP-binding, the interaction analysis of ADP-Mg^2+^-NLRP1-14^NACHT^ complexes was performed to infer the nature and mode of ADP-binding. Shown in S4 Fig, we noticed a similar mode of ADP-binding in most of the complexes and the crucial residues, ‘Lys’ (of Walker A), ‘Asp’ (first acidic residue of Walker B), ‘Pro’ (of PhhCW motif), and ‘His’ (of WH-His) [excluding ‘Arg’ of Sensor 1 motif] are found to be involved in ADP-Mg^2+^ coordination. In all, we observed a similar mode of ADP and ATP-binding in the NLRP1-14^NACHT^ models during dynamics. Further, our results on ADP-/ATP-Mg^2+^ interaction showed a good correlation with the previous predictions [28,29] and experimental findings [50–54].

### Opening-closing mechanism of NLRP^NACHT^ domain

**Fig 5:**
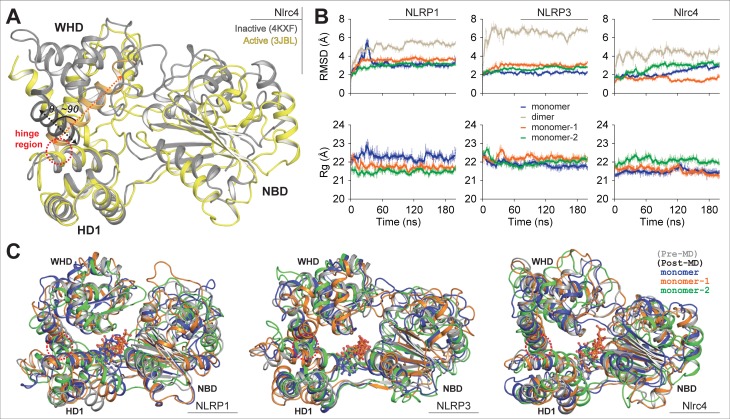
Overview of structural transitions facilitated by NLRP1/3/Nlrc4^NACHT^ models in response to ATP. (A) Superimposed view of open-close state of Nlrc4^NACHT^ domain; the inactive/closed structure is displayed in gray cartoon and the active/open state structure is presented in yellow cartoon; the hinge region is marked red circle. (B) Comparative view backbone RMSD and Rg of ATP-Mg^2+^ bound NACHT domains in monomeric and dimeric condition. (C) Superimposed view of NLRP/Nlrc4^NACHT^ models showed structural transitions (pre-MD structures are presented in grey cartoon and post MD structure in colored cartoon).

In NLRP sub-family, the ATPase activity/ATP-binding is well studied in NLRP1, NLRP3, NLRP7 and NLRP12 receptors [50–54]. The reports on NLRP1, NLRP3, and NLRP7 indicated that the binding of ATP is crucial for oligomerization and inflammasome activation [50–52]. However, a report on NLRP12 revealed that the recognition of ATP stimulates receptor’s anti-inflammatory response [53]. Till date, six experimental structures of NLRs (with NACHT domain) have been resolved; two closed structures (Nlrc4 (4KXF) [48] and *Oc*NOD2 (5IRL) [41]) and four open structures (Nlrc4 (3JBL, 5AJ2, and EMD-3139-3143) [55–57] and Nlrc4-NAIP5 complex (6B5B) [58]). Both the closed structures are ADP-bound while none of the open structures contain ATP-Mg^2+^ in their binding cavity. Recently, Hu *et al*. [55] and Zhang *et al*. [56] revealed that the active conformation of Nlrc4 has a rigid body movement (~90˚/87.5˚) of WHD-HD2-LRR module with respect to HD1 around the hinge region (between HD1 and WHD) (Fig 5A), which was later supported by Tenthorey *et al*. [58] in a recent report on Nlrc4-NAIP5 inflammasome complex. Conversely, Diebolder and co-authors suggested that the open conformation of Nlrc4 has 21˚ and 49˚ rigid body rotation of NBD-HD1 and LRR domain with respect to WHD-HD2 module (‘open lock’ structure) [57]; and no rigid body movement between HD1 and WHD. It was also reported that the ATP-hydrolysis (after ADP to ATP transition) might be the cause behind the conformational change of NLRs [49–53]. However, none of the reports describing the structural basis of inflammasome assembly [55–59], discussed the relevance of ATP-binding mechanism.

Hence, to infer the role of ATP-Mg^2+^ in the opening mechanism of NLRP^NACHT^ domains during dynamics, we calculated the radius of gyration (Rg) of whole protein and performed PCA of main chain atoms of apo and holo (ADP-/ATP-bound) conformers as a function of simulation time. As presented (in S5 Fig), we observed comparatively higher compactness and lower eigenvalue in ADP-bound complexes than that of ATP-bound ones. However, we could not notice significant structural changes in superimposed view of pre and post-MD structures of NLRP1-14^NACHT^-ATP-Mg^2+^ complexes (data not given). In a recent study, Afanasyeva *et al*. [59] reported the opening mechanism of ATP-bound TIP49 (AAA+ ATPase) during 30 ns of production runs. Conversely, in our previous study, we failed to observe the opening mechanism of ATP-bound NOD1/NOD2^NACHT^ domains in zebrafish model [41] even after 50 ns of MD simulation.

Amongst the NLRP-subfamily, NLRP1 and NLRP3 are well studied receptors and the experimental evidence revealed that the recognition of invading PAMPs followed by ATP-binding, orchestrate the multi-protein inflammasome complex [50,51]. Therefore, after noticing the importance of both the receptors and the role of long-range simulation in inspecting the molecular mechanisms of biological macromolecules [60], we simulated the NLRP1/3^NACHT^-ATP-Mg^2+^ bound complexes (along with Nlrc4^NACHT^-ATP-Mg^2+^ complex) over 200 ns timescale in monomeric as well as dimeric conformation [the homodimeric complexes were created by superimposing NBD-HD1 module of NLRP^NACHT^ models with the closed monomeric NACHT models with Nlrc4-NAIP5 inflammasome complex (6B5B) (see S6A Fig)]. After the final production runs, we calculated the backbone RMSD and Rg of the protein as a function of simulation time, and superimposed the pre- and post MD structures. Here, we noticed a stable backbone deviation and compactness in all the simulation systems (Fig 5B). Further, the superimposed structures revealed no significant structural changes as monomer after 200 ns of production run (Fig 5C). In homodimeric condition, we noticed a major structural changes in NLRP3^NACHT^ (S6B Fig); however, the superimposed view of both the monomer subunits (pre- and post-MD) showed no significant rigid body movement between HD1 and WHD (Fig 5C). Here it can be speculated that the interaction of ATP-Mg^2+^ might not be the only component that drive the switching mechanism in NLRs.

Among NLRPs, NLRP3 is the well-studied receptor and one of the significant contributors to diverse inflammatory diseases [61]. In recent scenario, the exploration of inflammasome and development of novel therapeutics for its regulation has gained the momentum [62]. Though several efforts have been made in this direction for the alienation of inflammasome activity by manipulating the signaling pathways [63], the in-depth inhibitory mechanism remains unresolved yet. Until this day, some inhibitors have been proposed for the regulation of uncontrolled inflammasome activity [64–67]. In AAA+/P-loop protein family, the ATP-binding pocket (resides in the kinase domain) have been widely studied for the development of structure-guided small molecule inhibitors [68]. In this context, the proposed ATP-binding cavity will definitely assist in designing novel inhibitors for the regulation of uncontrolled inflammasome activity.

The 3D model structure of macromolecules are essential entities to understand the mechanistic details of certain biological functions. Several experimental methods such as X-ray crystallography, Cryo-EM, NMR and SAXS provide atomistic details of the targeted macromolecules. However, the above methods has their own limitations and in general, the dynamic activity of complex bio-macromolecular systems are difficult to study. Thus, the theoretical approaches that include template-assisted molecular modeling, molecular docking, and molecular dynamics simulations are developed for the better understanding of biomolecular machinery and increasingly used to bridge this gap. In recent days, such computational methods have contributed significantly towards the study of the structural and functional aspects of several NLR proteins [69–75] and the protein of interest in this study. In 2012, Zurek et al. demonstrated the distinct activation mechanism of NOD1 and NOD2^NACHT^ and revealed that the mutation of WH-His has contrasting effect on NF-κB signaling mechanism [54]. As an example, using such methods, we have hypothesized a differential mode of ATP-binding in NOD1 and NOD2^NACHT^ domain (in zebrafish model) and suggested that differential mode of ATP-binding might be one of the causes behind the distinct activation mechanism [40]. In contrast to our prediction, Maekawa et al. revealed a common mode of ADP-binding in the *Oc*NOD2 structure [41] as observed in the Nlrc4-ADP complex crystal structure [48]. In a recent publication, we have reported that NOD1^CARD^ possibly is recruiting multiple interfaces for RIP2-mediated CARD-CARD interaction using theoretical approaches [40]. In the report, we have proposed the ‘type-I’ homodimeric models for both NOD1 and RIP2 CARDs and also notified the possible existence of ‘type-III’ RIP2^CARD^ homodimer in 1:2 NOD1:RIP2 heterotrimeric CARD:CARD complex [74]. Recent reports on RIP2^CARD^ filamentous structure demonstrated that RIP2^CARD^ uses all its interfaces (type-I, II and III) for filament formation [76,77], which is found to be consistent with our observation on RIP2^CARD^ homodimer formation [74]. The above discussion implies that though such approaches have positive directions in investigating the unexplored molecular mechanisms regulated by biological macromolecules; one should not overlook their limitations and pitfalls, particularly issues on force fields, time margins, and quantum effect.

## Conclusion

Due to the lack of sufficient experimental structural data, the role ATP-binding in opening-closing mechanism of NLRs is not fully understood. More so, the active conformations of Nlrc4 could not quite elucidate the role of ATP in molecular switching activity [55–58]. Henceforth, an attempt has been made to illustrate the structural, functional and dynamic aspects of ATP-binding in NLRP^NACHT^ domains adopting the structural bioinformatics approaches. Our findings indicated a common mode of ATP binding; where the γ-phosphate was buried inside the core cavity and the adenine ring posed outward. Further, the long-range MD simulation indicated that ATP-Mg^2+^-binding might not be the key factors for the switching mechanism of NLRs. Here, it can be speculated that the PAMP recognition might have the major role behind the switching mechanism in NLRs. In conclusion, the findings of this study will facilitate in comprehending the molecular mechanism governed by NLRPs. Further, the projected ATP-binding pocket will be greatly useful in designing novel NACHT inhibitors for regulating the receptor-specific signaling mechanisms.

## Supporting information

S1 Table Overview of NLRP-subfamily according to their domain distribution and key ADP-/ATP-Mg^2+^ binding motifs (key residues are in bold font). (DOC)

S2 Table Properties of the simulation systems. (DOC)

S3 Table Model validation report of NLRP1-14^NACHT^ 3D models. (DOC)

S4 Table AutoDock Vina molecular docking scores of ADP/ATP-NLRP^NACHT^ complexes. (DOC)

S1 Fig Illustrations of NLRPNACHT-ADP/ATP/Mg2+ conformations generated via AutoDock Vina (A) and (B) manual docking. ATP is shown in ball-stick model, protein in cartoon, and Mg2+ ions are displayed in green ball model.
(TIF)

S2 Fig Structural overview of ADP and ATP binding orientation before and after MD simulation; the proteins are displayed in cartoon (grey cartoon, pre-MD structure and colored cartoon, post-MD structure) and ATP in stick model. (TIF)

S3 Fig Illustration of inter-residual distances between ATP and conserved key residues of respective NLRPs, those are not found interacting with in 5 Å of ATP. (TIF)

S4 Fig Overview of detailed intermolecular interactions governed by ADP and NLRPNACHT models. The 3D models are displayed in cartoon; ADPs are visualized in light green ball-sticks and the residues interacting within 5Å of ATP are visualized in white stick model. The black dotted lines represent the intermolecular polar contacts. The key interacting residues are presented in bold font and colored based on their physicochemical parameters.
(TIF)

S5 Fig (A) Radius of gyration and (B) Eigenvalues of the all NLRPNACHT models in apo, ADP and ATP-bound conditions.
(TIF)

S6 Fig (A) Structural overview of Nlrc4 homodimer (6B5B) (open structure). Functional domain regions are presented in colored cartoons (green, LRR; orange HD2; red, NACHT) (B) Superimposed view of closed NLRP1, NLRP3 and Nlrc4NACHT models with the Nlrc4 open structure. (C) Pre- and Post MD superimposed structural overview of respective homodimers. The pre-MD structures are visualized in dark-gray cartoon and post-MD structures are in white (NLRP1), blue (NLRP3) and yellow (Nlrc4).
(TIF)
