# Supplementary material for: Deciphering the ATP-binding mechanism(s) in NLRP-NACHT 3D models using structural bioinformatics approaches
Source: PLoS One. 2018 Dec 20;13(12):e0209420. doi: 10.1371/journal.pone.0209420 (PMC6301626; doi:10.1371/journal.pone.0209420)
Supplement: S1 Table — (DOC) [file pone.0209420.s001.doc]

S1 Table. Overview of NLRP-subfamily according to their domain distribution and key ADP-/ATP-Mg^2+^ binding motifs (key residues are in bold font)

| NLRs | Accession numbers | Length | PYD | NACHT | ATP/ Nucleotide-binding motifs | | | | | HD2 | LRR region  (in numbers) |
| --- | --- | --- | --- | --- | --- | --- | --- | --- | --- | --- | --- |
|  |  |  |  |  | Walker A | Walker B | Sensor 1 | PxhCW | WH-His |  |  |
| NLRP1* | NP_127497 | 1473 | 1-90 | 328-637 | GAAGIG**K**S | FIL**D**GVDE | TA**R** | **P**WVSW | FI**H** | 649-758 | 809-976 (6) |
| NLRP2 | NP_060322 | 1062 | 1-92 | 207-517 | GPAGLG**K**T | FVI**D**GFDE | TT**R** | **P**AVCW | FI**H** | 531-644 | 756-1035 (10) |
| NLRP3 | NP_004886 | 1036 | 1-93 | 220-536 | GAAGIG**K**T | FLM**D**GFDE | TT**R** | **P**LVCW | FI**H** | 554-670 | 742-1023 (10) |
| NLRP4 | NP_604393 | 994 | 1-92 | 149-466 | GPQGIG**K**T | FVI**D**SFEE | AI**K** | **P**LLCW | FL**H** | 477-593 | 639-975 (12) |
| NLRP5 | NP_703148 | 1200 | 55-146 | 280-598 | GKSGIG**K**S | FII**D**GFDD | TV**R** | **P**AVGS | FF**H** | 612-727 | 782-1176 (14) |
| NLRP6 | NP_612202 | 892 | 12-101 | 196-513 | GPAGIG**K**T | FIL**D**GADE | TT**R** | **P**FVCW | FI**D** | 522-640 | 729-868 (5) |
| NLRP7 | NP_996611 | 980 | 1-91 | 178-482 | GPAGVG**K**T | FVV**D**GLDE | TT**R** | **P**AVCW | FI**H** | 496-611 | 676-955 (10) |
| NLRP8 | NP_789781 | 1048 | 31-121 | 204-521 | GAPGIG**K**T | LLL**D**GFEE | MI**R** | **P**VVCW | FT**L** | 534-649 | 704-1006 (11) |
| NLRP9 | NP_789790 | 991 | 1-92 | 146-459 | GPDGIG**K**T | FIM**D**GFEQ | AL**G** | **P**FTCW | FM**H** | 470-585 | 631-967 (12) |
| NLRP10 | NP_789791 | 655 | 3-94 | 167-480 | GSAGTG**K**T | FIL**D**GFDE | TT**R** | **P**GICW | FR**H** | 491-604 | - |
| NLRP11 | NP_659444 | 1033 | 1-89 | 147-464 | GERASG**K**T | FIL**E**DLDN | SS**R** | **A**ILCW | FI**H** | 471-585 | 634-971 (12) |
| NLRP12 | NP_653288 | 1061 | 3-93 | 211-528 | GAAGIG**K**S | FII**D**GFDE | TT**R** | **P**LVCW | FI**H** | 537-654 | 714-1052 (12) |
| NLRP13 | NP_789780 | 1043 | 7-105 | 229-550 | GRAGVG**K**T | FII**D**GFEE | TI**K** | **P**MVCW | FT**H** | 561-678 | 683-1033 (11) |
| NLRP14 | NP_789792 | 1093 | 5-95 | 177-484 | GAAGVG**K**T | FII**D**SFDE | TT**R** | **P**LVCW | FT**H** | 507-622 | 674-1068 (14) |

*Additional domains of NLRP1: FIIND and CARD.
