# Supplementary material for: Deciphering the ATP-binding mechanism(s) in NLRP-NACHT 3D models using structural bioinformatics approaches
Source: PLoS One. 2018 Dec 20;13(12):e0209420. doi: 10.1371/journal.pone.0209420 (PMC6301626; doi:10.1371/journal.pone.0209420)
Supplement: S2 Table — (DOC) [file pone.0209420.s002.doc]

S2 Table. Properties of the simulation systems.

| Simulation system | Simulation state | Total no. of atoms | Time (ns) |
| --- | --- | --- | --- |
| NLRP1 | Apo | 88852 | 50 |
|  | ADP^AD^ | 88849 | 50 |
|  | ADP^MD^ | 88891 | 50 |
|  | ATP^AD^ | 88858 | 50 |
|  | ATP^MD^ | 88888 | 50 |
| NLRP2 | Apo | 72811 | 50 |
|  | ADP^AD^ | 72816 | 50 |
|  | ADP^MD^ | 72759 | 50 |
|  | ATP^AD^ | 72809 | 50 |
|  | ATP^MD^ | 72746 | 50 |
| NLRP3 | Apo | 80720 | 50 |
|  | ADP^AD^ | 80716 | 50 |
|  | ADP^MD^ | 80701 | 50 |
|  | ATP^AD^ | 80721 | 50 |
|  | ATP^MD^ | 80709 | 50 |
| NLRP4 | Apo | 76311 | 50 |
|  | ADP^AD^ | 76247 | 50 |
|  | ADP^MD^ | 76223 | 50 |
|  | ATP^AD^ | 76249 | 50 |
|  | ATP^MD^ | 76225 | 50 |
| NLRP5 | Apo | 80537 | 50 |
|  | ADP^AD^ | 80555 | 50 |
|  | ADP^MD^ | 80567 | 50 |
|  | ATP^AD^ | 80549 | 50 |
|  | ATP^MD^ | 80576 | 50 |
| NLRP6 | Apo | 74215 | 50 |
|  | ADP^AD^ | 74230 | 50 |
|  | ADP^MD^ | 74287 | 50 |
|  | ATP^AD^ | 74224 | 50 |
|  | ATP^MD^ | 74296 | 50 |
| NLRP7 | Apo | 76506 | 50 |
|  | ADP^AD^ | 76521 | 50 |
|  | ADP^MD^ | 76614 | 50 |
|  | ATP^AD^ | 76521 | 50 |
|  | ATP^MD^ | 76623 | 50 |
| NLRP8 | Apo | 74860 | 50 |
|  | ADP^AD^ | 74850 | 50 |
|  | ADP^MD^ | 74889 | 50 |
|  | ATP^AD^ | 74855 | 50 |
|  | ATP^MD^ | 74885 | 50 |
| NLRP9 | Apo | 80292 | 50 |
|  | ADP^AD^ | 80415 | 50 |
|  | ADP^MD^ | 80298 | 50 |
|  | ATP^AD^ | 80418 | 50 |
|  | ATP^MD^ | 80301 | 50 |
| NLRP10 | Apo | 88751 | 50 |
|  | ADP^AD^ | 88754 | 50 |
|  | ADP^MD^ | 88775 | 50 |
|  | ATP^AD^ | 88745 | 50 |
|  | ATP^MD^ | 88799 | 50 |
| NLRP11 | Apo | 74971 | 50 |
|  | ADP^AD^ | 74998 | 50 |
|  | ADP^MD^ | 74998 | 50 |
|  | ATP^AD^ | 74994 | 50 |
|  | ATP^MD^ | 75006 | 50 |
| NLRP12 | Apo | 76771 | 50 |
|  | ADP^AD^ | 76756 | 50 |
|  | ADP^MD^ | 76768 | 50 |
|  | ATP^AD^ | 76764 | 50 |
|  | ATP^MD^ | 76770 | 50 |
| NLRP13 | Apo | 80164 | 50 |
|  | ADP^AD^ | 80202 | 50 |
|  | ADP^MD^ | 80178 | 50 |
|  | ATP^AD^ | 80204 | 50 |
|  | ATP^MD^ | 80180 | 50 |
| NLRP14 | Apo | 80076 | 50 |
|  | ADP^AD^ | 80072 | 50 |
|  | ADP^MD^ | 80099 | 50 |
|  | ATP^AD^ | 80074 | 50 |
|  | ATP^MD^ | 80110 | 50 |
| Nlrc4 | Apo | 74807 | 50 |
|  | ADP | 74792 | 50 |
|  | ATP^MD^ | 74888 | 50 |

^AD^: AutoDock; ^MD^: Manually docked

| Nlrc4-ATP | monomer | 80774 | 200 |
| --- | --- | --- | --- |
|  | dimer | 168170 |
| NLRP1-ATP | monomer | 98121 | 200 |
|  | dimer | 178963 |
| NLRP3-ATP | monomer | 96086 | 200 |
|  | dimer | 173718 |
