# Supplementary material for: Deciphering the ATP-binding mechanism(s) in NLRP-NACHT 3D models using structural bioinformatics approaches
Source: PLoS One. 2018 Dec 20;13(12):e0209420. doi: 10.1371/journal.pone.0209420 (PMC6301626; doi:10.1371/journal.pone.0209420)
Supplement: S3 Table — (DOC) [file pone.0209420.s003.doc]

S3 Table. Model validation report of NLRP1-14^NACHT^ 3D models

| Protein models | PROCHECK (Ramachandran Plot) | | | | | Verify3D | ProSA |
| --- | --- | --- | --- | --- | --- | --- | --- |
|  | Most favored (%) | Additional allowed (%) | Generously allowed (%) | Disallowed (%) | Overall G-factor | 1D-3D score (%) | Z-score |
| NLRP1 | 91.6 | 7.3 | 0.7 | 0.4 | 0.26 | 88.39 | -7.76 |
| NLRP2 | 91.3 | 7.6 | 0.4 | 0.7 | 0.25 | 91.32 | -8.17 |
| NLRP3 | 91.6 | 7.3 | 0.3 | 0.7 | 0.25 | 88.64 | -6.62 |
| NLRP4 | 86.6 | 10.2 | 0.4 | 2.8 | 0.21 | 81.76 | -7.98 |
| NLRP5 | 90.5 | 7.8 | 0.7 | 1.1 | 0.22 | 85.27 | -7.77 |
| NLRP6 | 89.1 | 8.8 | 1.5 | 0.7 | 0.23 | 82.08 | -7.81 |
| NLRP7 | 91.2 | 6.9 | 0.7 | 1.1 | 0.23 | 94.86 | -7.93 |
| NLRP8 | 89.8 | 8.5 | 1.1 | 0.7 | 0.23 | 84.28 | -6.83 |
| NLRP9 | 87.2 | 9.3 | 1.0 | 2.4 | 0.32 | 89.81 | -6.61 |
| NLRP10 | 89.4 | 7.4 | 1.4 | 1.8 | 0.30 | 87.58 | -6.25 |
| NLRP11 | 88.7 | 9.6 | 0.7 | 1.0 | 0.40 | 84.28 | -6.51 |
| NLRP12 | 90.7 | 7.5 | 0.4 | 1.4 | 0.20 | 87.11 | -7.47 |
| NLRP13 | 88.2 | 9.1 | 0.3 | 2.4 | 0.33 | 86.96 | -7.66 |
| NLRP14 | 86.0 | 11.3 | 1.0 | 1.7 | 0.23 | 83.65 | -7.22 |
