# Supplementary material for: Deciphering the ATP-binding mechanism(s) in NLRP-NACHT 3D models using structural bioinformatics approaches
Source: PLoS One. 2018 Dec 20;13(12):e0209420. doi: 10.1371/journal.pone.0209420 (PMC6301626; doi:10.1371/journal.pone.0209420)
Supplement: S4 Table — (DOC) [file pone.0209420.s004.doc]

S4 Table. AutoDock Vina molecular docking scores of ADP/ATP-NLRP^NACHT^ complexes

| Proteins | ADP | | ATP | |
| --- | --- | --- | --- | --- |
|  | Binding energy (kcal/mol) | No. of H-bonds | Binding energy (kcal/mol) | No. of H-bonds |
| NLRP1 | -7.5 | 5 | -7.6 | 5 |
| NLRP2 | -8.4 | 3 | -8.6 | 8 |
| NLRP3 | -6.4 | 3 | -7.0 | 7 |
| NLRP4 | -7.1 | 4 | -8.0 | 4 |
| NLRP5 | -8.9 | 4 | -8.4 | 5 |
| NLRP6 | -5.8 | - | -5.6 | 3 |
| NLRP7 | -8.6 | 5 | -8.3 | 7 |
| NLRP8 | -7.1 | 1 | -7.3 | 5 |
| NLRP9 | -6.4 | 2 | -6.7 | 3 |
| NLRP10 | -8.0 | 1 | -8.3 | 6 |
| NLRP11 | -7.1 | 2 | -7.3 | 4 |
| NLRP12 | -8.1 | 5 | -8.4 | 8 |
| NLRP13 | -8.1 | 6 | -7.1 | 1 |
| NLRP14 | -8.6 | 6 | -8.4 | 4 |
