# Supplementary figures and images for: Deciphering the ATP-binding mechanism(s) in NLRP-NACHT 3D models using structural bioinformatics approaches
Source: PLoS One. 2018 Dec 20;13(12):e0209420. doi: 10.1371/journal.pone.0209420 (PMC6301626; doi:10.1371/journal.pone.0209420)

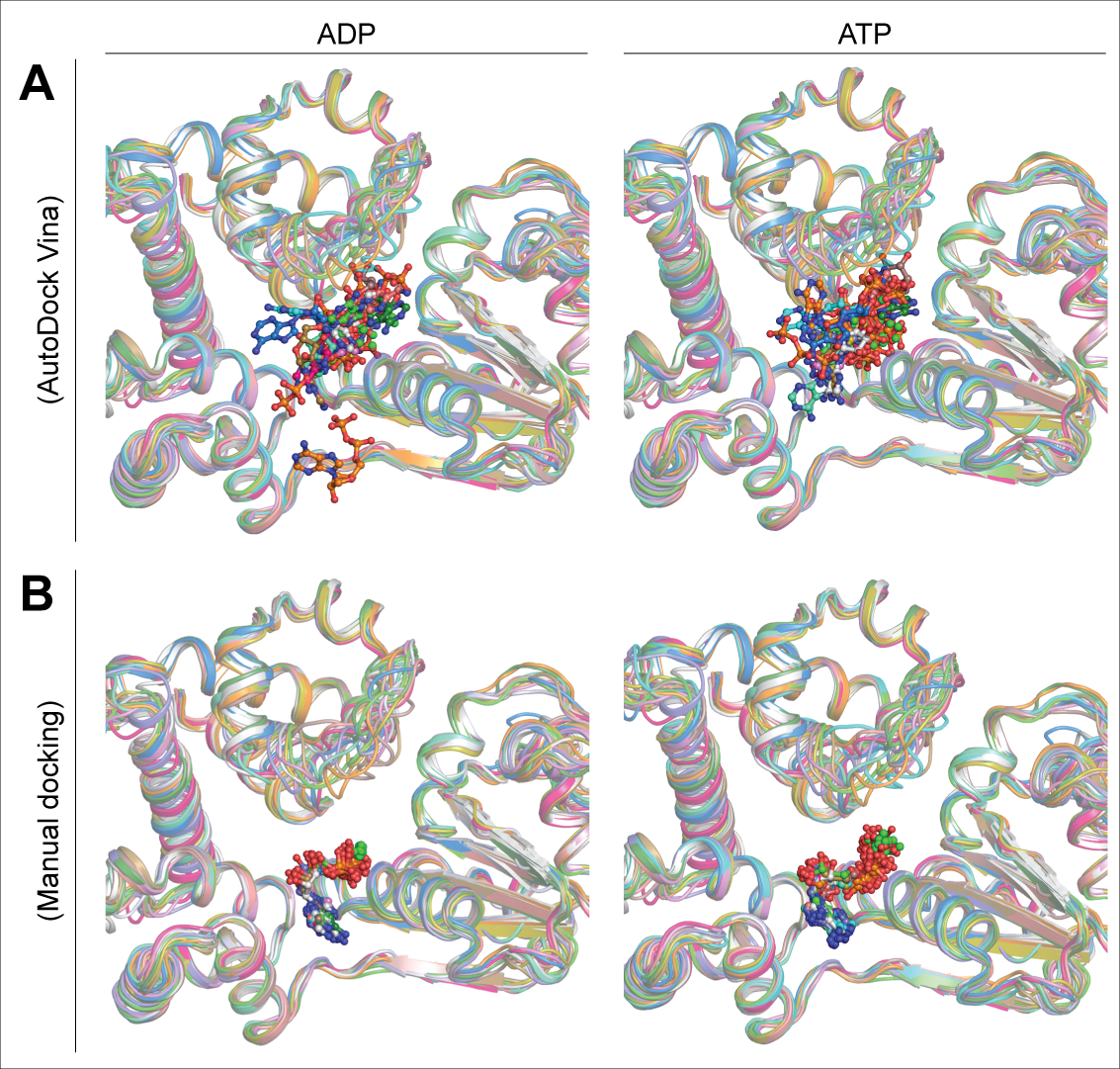

Supplement: S1 Fig — Illustrations of NLRPNACHT-ADP/ATP/Mg2+ conformations generated via AutoDock Vina (A) and (B) manual docking. ATP is shown in ball-stick model, protein in cartoon, and Mg2+ ions are displayed in green ball model. (TIF) [file pone.0209420.s005.tif]

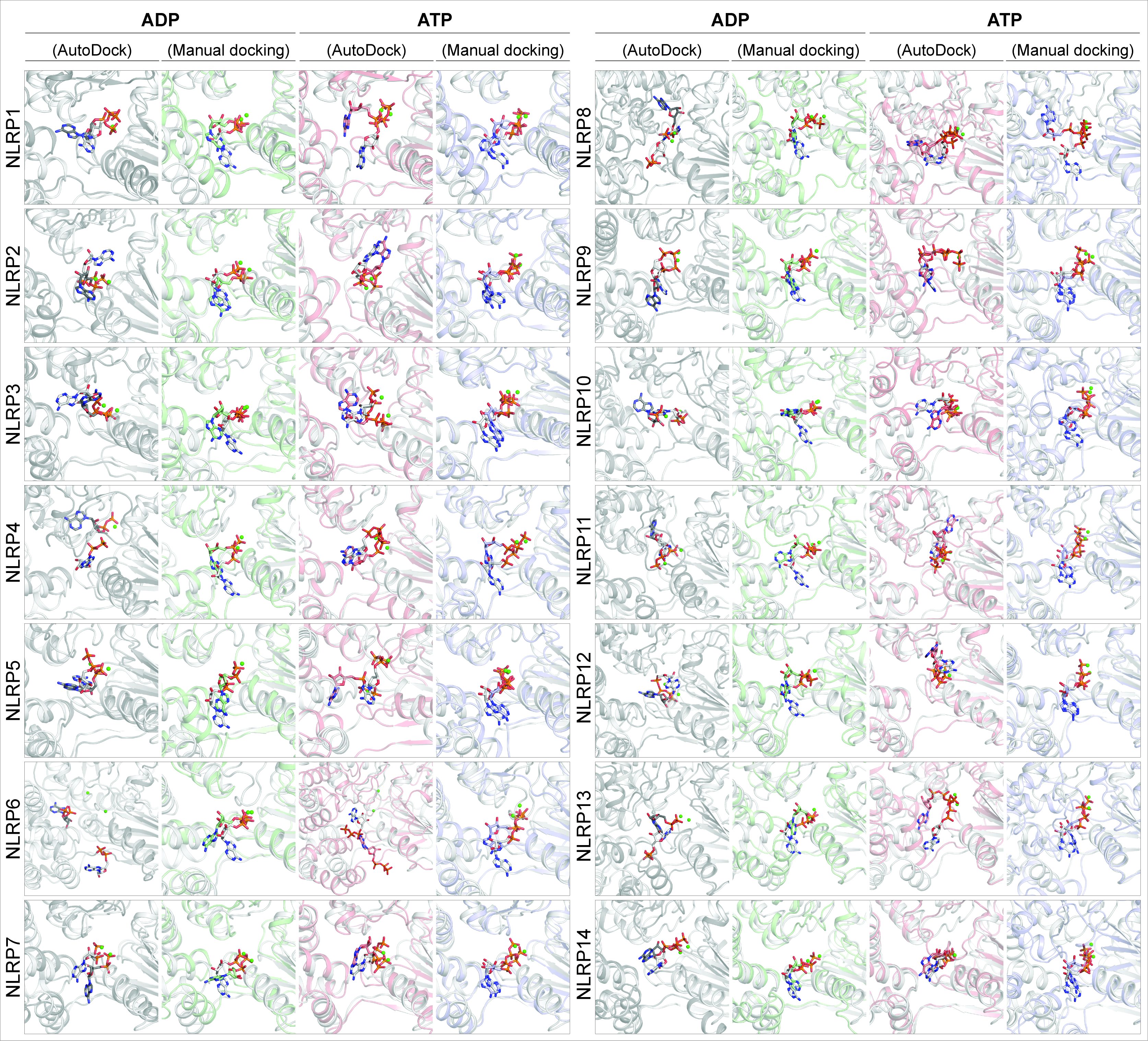

Supplement: S2 Fig — (TIF) [file pone.0209420.s006.tif]

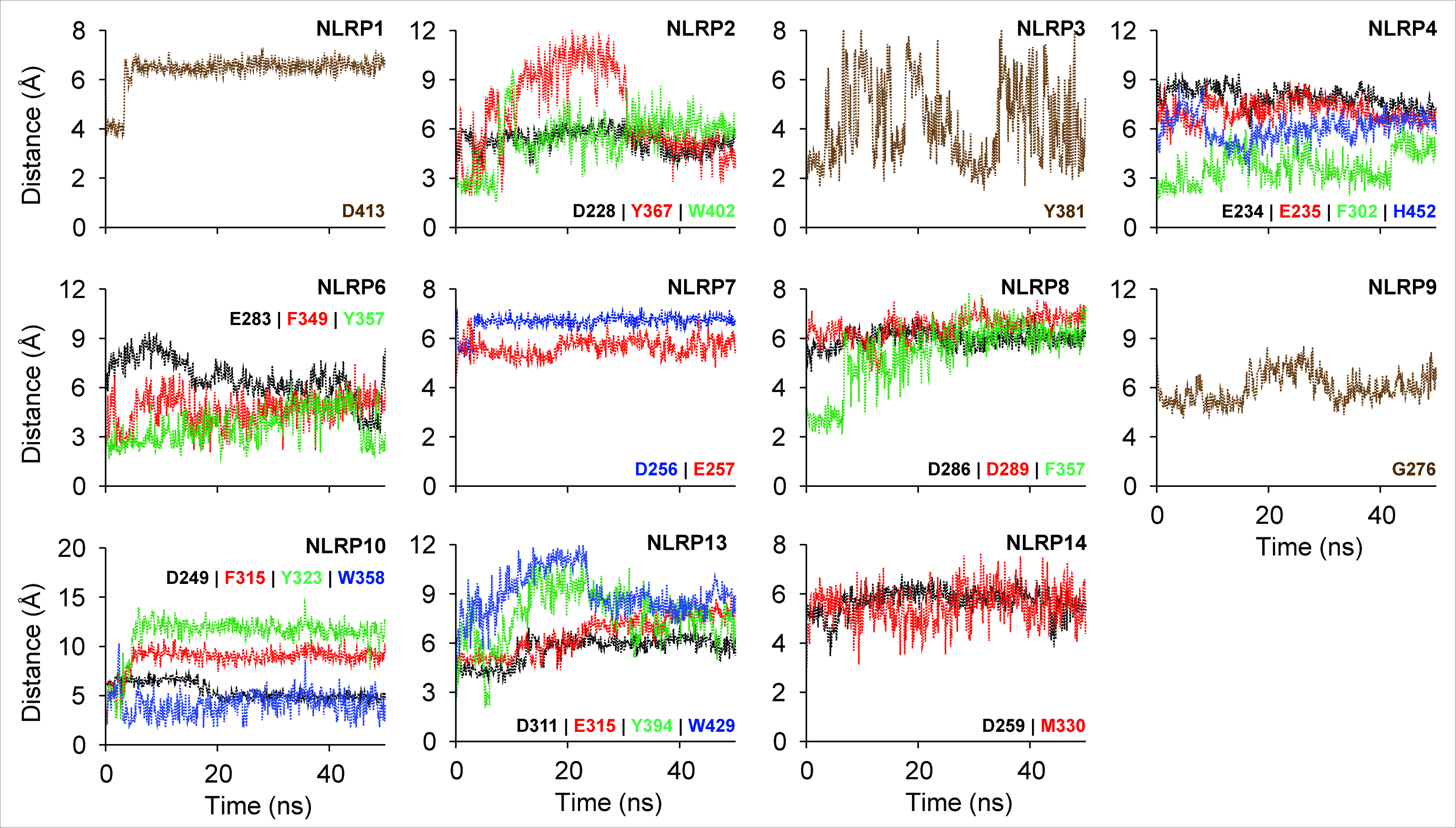

Supplement: S3 Fig — (TIF) [file pone.0209420.s007.tif]

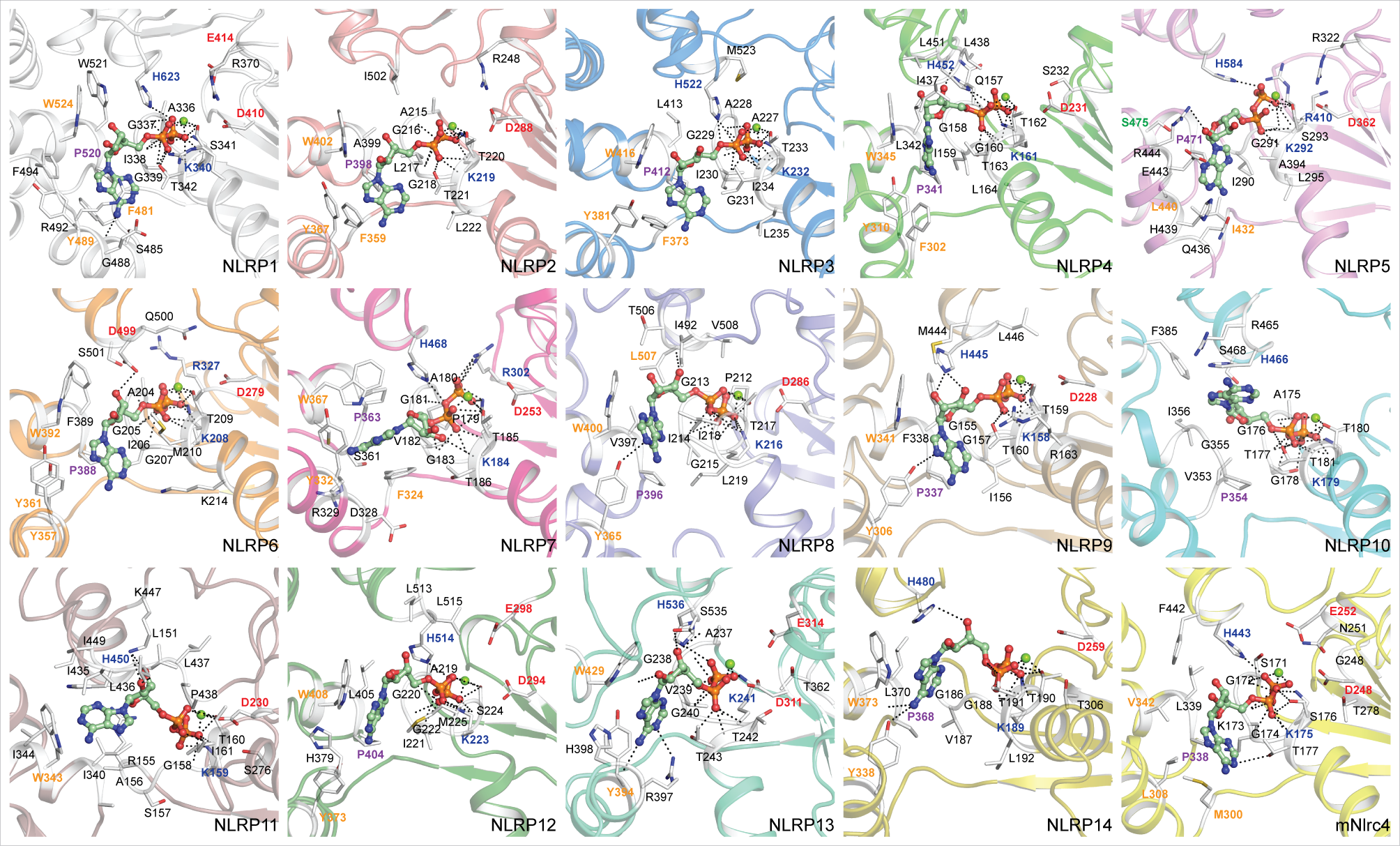

Supplement: S4 Fig — The 3D models are displayed in cartoon; ADPs are visualized in light green ball-sticks and the residues interacting within 5Å of ATP are visualized in white stick model. The black dotted lines represent the intermolecular polar contacts. The key interacting residues are presented in bold font and colored based on their physicochemical parameters. (TIF) [file pone.0209420.s008.tif]

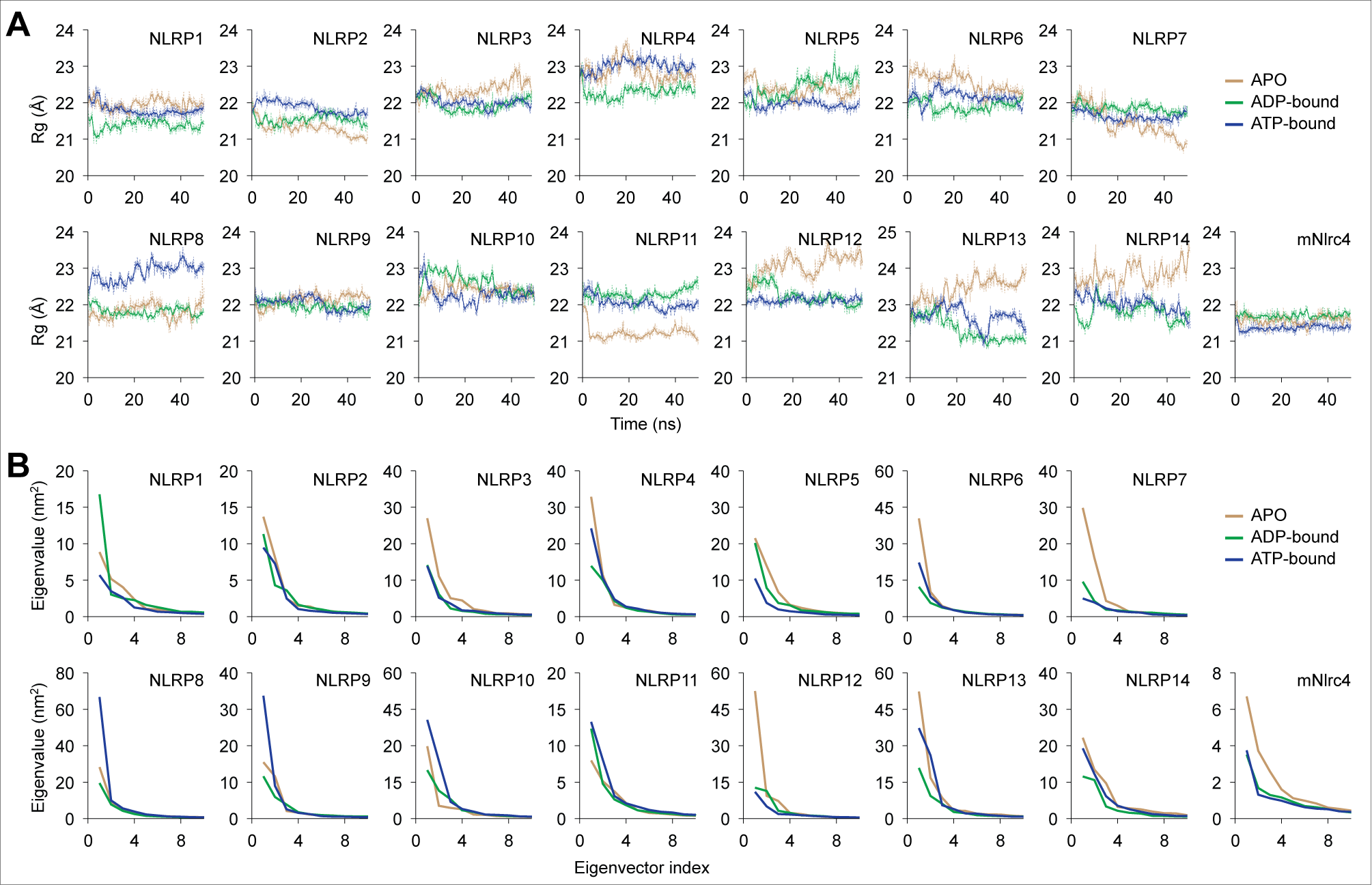

Supplement: S5 Fig — (A) Radius of gyration and (B) Eigenvalues of the all NLRPNACHT models in apo, ADP and ATP-bound conditions. (TIF) [file pone.0209420.s009.tif]

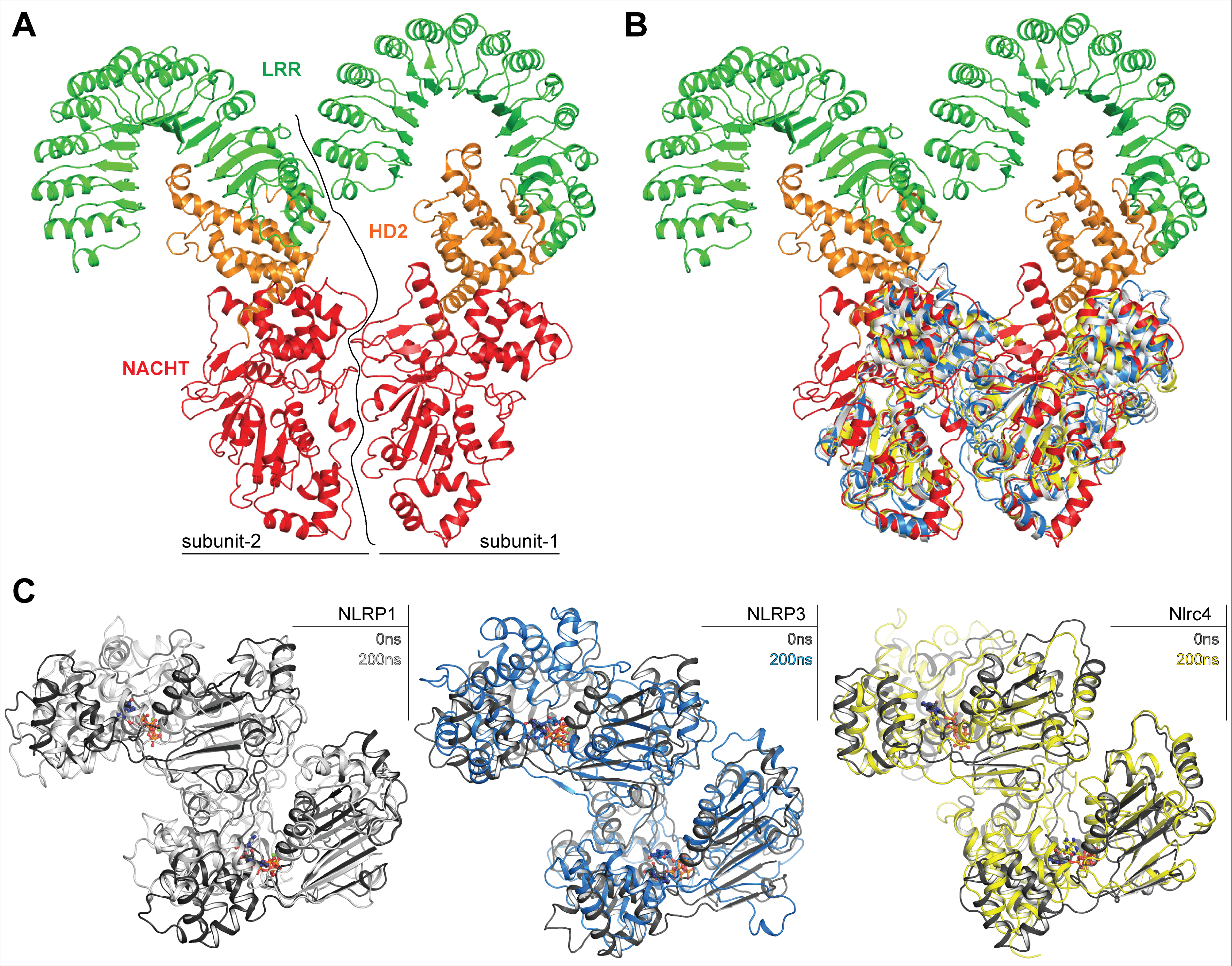

Supplement: S6 Fig — (A) Structural overview of Nlrc4 homodimer (6B5B) (open structure). Functional domain regions are presented in colored cartoons (green, LRR; orange HD2; red, NACHT) (B) Superimposed view of closed NLRP1, NLRP3 and Nlrc4NACHT models with the Nlrc4 open structure. (C) Pre- and Post MD superimposed structural overview of respective homodimers. The pre-MD structures are visualized in dark-gray cartoon and post-MD structures are in white (NLRP1), blue (NLRP3) and yellow (Nlrc4). (TIF) [file pone.0209420.s010.tif]
